# Optimized Parameters of Diffusion-Weighted MRI for Prediction of the Response to Neoadjuvant Chemoradiotherapy for Locally Advanced Rectal Cancer

**DOI:** 10.1155/2019/9392747

**Published:** 2019-10-13

**Authors:** Jie Li, Jia Wang, Jing Pang, Shougen Cao, Jingjing Chen, Wenjian Xu

**Affiliations:** ^1^Department of Radiology, The Affiliated Hospital of Qingdao University, Qingdao, Shandong, China; ^2^Department of Ultrasound, Qingdao Women and Children Hospital, Qingdao, Shandong, China; ^3^Department of General Surgery, The Affiliated Hospital of Qingdao University, Qingdao, Shandong, China

## Abstract

**Aim:**

To identify the optimal diffusion-weighted MRI-derived parameters for predicting the response to neoadjuvant chemoradiotherapy in locally advanced rectal cancer.

**Methods:**

This prospective study enrolled 92 patients who underwent neoadjuvant chemoradiotherapy. Diffusion-weighted MRI sequences with two *b*-value combinations of *b* (0, 800) and *b* (0, 1000) were acquired before the start of neoadjuvant chemoradiotherapy and surgery. The pathological tumor regression grade was obtained according to the Mandard criteria, recommended by the seventh edition of the American Joint Committee on Cancer, to act as the reference standard. Pathological good responders (pathological tumor regression grade 1-2) were compared with poor responders (pathological tumor regression grade 3–5).

**Results:**

The good responder group contained 37 (40.2%) patients and the poor responder group 55 (59.8%) patients. Both before and after neoadjuvant chemoradiotherapy, the mean ADC value for *b* = 1000 was significantly higher than that for *b* = 800. In the two patient groups, the post-ADC value and ΔADC for *b* = 800 were significantly lower than those for *b* = 1000, but percentages of ADC increase for *b* = 800 and *b* = 1000 showed no significant difference.

**Conclusions:**

The percentage of ADC increase, as an optimized predictor unaffected by different *b*-values, may have a significant role in differentiating those patients with a good response to N-CRT from those with a poor response.

## 1. Introduction

Total mesorectal excision (TME) and neoadjuvant chemoradiotherapy (N-CRT) have dramatically improved the clinical outcomes of locally advanced rectal cancer (LARC) [1], and it has been reported that local recurrence rates have dropped from 30% to less than 10% [2]. Additionally, approximately 10%–30% of patients with LARC show a pathological complete response (pCR) after N-CRT [3, 4].

Several studies have shown that for patients with LARC showing a good response to N-CRT, the N-CRT course should be increased appropriately to improve the chance of obtaining a pCR [4–7]. Although there are arguments on “wait-and-see” policy for patients with pCR [6], a pCR suggests less invasive treatment regimens such as a local excision or a potential “wait-and-see” strategy with careful follow-up [8, 9]. An accurate method to preoperatively assess the response to N-CRT is therefore essential to facilitate a precise patient-tailored treatment for LARC.

As a noninvasive imaging method, MRI is useful for assessment of the response to therapy [10, 11]. Additionally, diffusion-weighted imaging (DWI) and the DWI-derived apparent diffusion coefficient (ADC) can be used for quantitative analysis of the intratumoral changes in tumorous cellular density and extracellular space induced by N-CRT [12, 13].

Published articles revealed that DWI-derived parameters, such as ADC values after N-CRT, changes in ADC value, and percentage of ADC increase, are useful for assessing the response to N-CRT [14–17]. In these studies, *b*-value combinations of (0, 800) or (0, 1000) were typically used to perform DWI. In clinical settings, these two *b*-value combinations are considered acceptable and reasonable. However, it was reported that ADC values varied greatly with different *b*-value combinations [18, 19]. There were limited articles about the impact of ADC values measured through ADC map using two different *b*-value combinations on assessment of response to N-CRT.

Therefore, the purpose of this study was to identify the optimal diffusion-weighted MRI-derived parameters for discriminating between good and poor responders to N-CRT, as well as to investigate the diagnostic performance of ADC-based measurements in predicting the response to N-CRT.

## 2. Materials and Methods

### 2.1. Patients

This prospective study was reviewed by the ethics committee of our institution, and informed consent was obtained from all patients. Patients with LARC who received N-CRT between September 2015 and January 2019 were included in the study. All patients underwent surgery within 6–7 weeks after the end of N-CRT. The criteria for admission of patients were as follows: (1) rectal adenocarcinoma confirmed by biopsy and rectoscopy in the initial diagnosis of rectal cancer; (2) stage T3/T4 locally advanced rectal cancer with or without peripheral lymph node metastasis; (3) no evidence of distant metastasis; and (4) no antitumor therapy before receiving N-CRT. The exclusion criteria included the following: (1) patients with rectal cancer whose N-CRT was incomplete for any reason; (2) incomplete MRI or clinical data; and (3) MR images that were difficult to evaluate because of factors such as motion or metal artifacts.

### 2.2. N-CRT Project

All patients were treated with long-term chemoradiotherapy that included the following:

1. Radiotherapy plan: 3D-conformational multiple field technique with a total dose of 45 Gy, with a daily radiation dose of 1.8 Gy delivered 5 days a week for 5 weeks
2. Chemotherapy regimen: weekly intravenous injection of 60 mg/m^2^ oxaliplatin and daily oral administration of 825 mg/m^2^ capecitabine

### 2.3. Imaging Techniques

MRI examinations were performed using a 3.0 T MRI scanner (Signa; GE Medical Systems, Milwaukee, WI). All patients underwent an MR examination in the week before they received N-CRT and again 3 days before their operation. The MRI acquisitions and parameters included the following: (1) T2-weighted imaging (T2WI) in axial, coronal, and sagittal orientations using a fast spin echo (FSE) sequence with repetition time/echo time (TR/TE):5600/90 ms, slice thickness: 2 mm, interval: 0 mm, and field of view (FOV): 200 × 200 mm (axial) and 200 × 400 mm (sagittal) and (2) axial diffusion-weighted imaging using a spin-echo echo-planar imaging sequence (SE-EPI sequence), with TR/TE: 5000/70.6 ms, FOV: 420 × 420 mm, and diffusion gradients of *b* (0, 800) s/mm^2^ and *b* (0, 1000) s/mm^2^.

### 2.4. Image Analysis

All images were evaluated on an ADW4.3 postprocessing workstation (GE Medical Systems). Two radiologists with 7 and 10 years of abdominal MRI experience performed the image processing. The ADC map was used to measure the ADC value in order to avoid the T2 shine-through effect. DWI and T2WI were used to determine the tumor boundaries, with the two radiologists reaching an agreement on the area of the regions of interest (ROIs). A single-slice ROI was used to measure the ADC value of a tumor according to the following steps: select a maximum cross-sectional slice of the lesion, delineate the entire range of the tumor, measure the ADC value three times, and then calculate the mean ADC value. If there was no visible residual tumor on MR imaging after N-CRT, the ROI was placed on the residual rectum in the same area that was used in the initial MR imaging before N-CRT. ADC change was calculated according to the formula: ΔADC = ADC value after N-CRT (post-ADC) − ADC value before N-CRT (pre-ADC). The percentage of ADC increase after treatment was calculated using the formula percentage of ADC increase = (post-ADC − pre-ADC)/pre-ADC × 100%.

### 2.5. Histopathological Evaluation

Pathological evaluation is the gold standard for evaluating the response to N-CRT. Pathological reports were made by two pathologists, and pathological tumor regression grading (pTRG) was evaluated according to the guidelines of the Mandard criteria recommended by the seventh edition of the American Joint Committee on Cancer (AJCC). If the scores of the two pathologists were inconsistent, a consensus was adopted. The Mandard standard TRG score and efficacy evaluation criteria are defined as the following: (1) pTRG1 refers to absence of residual tumor tissue on pathological sections and the intestinal wall where the original tumor was located shows fibrotic changes; (2) pTRG2 refers to scattered residual tumor cells in fibrotic tissue of the tumors; (3) pTRG3 refers to fibrosis, although there are many residual tumor cells in the tumors; (4) pTRG4 refers to residual tumor cells that significantly exceed the fibrotic range; and (5) pTRG5 means that there is no obvious effect of radiotherapy and chemotherapy. pTRG1 is considered as pCR and pTRG2–5 as no pCR. In this study, enrolled patients were divided into two groups according to histopathologic tumor regression grade following the methods of previous articles: good responders (pTRG1-2) and poor responders (pTRG3–5).

### 2.6. Statistical Analysis

The *χ*^2^ test was used to compare patient characteristics between good responder and poor responder groups. Independent sample *t*-tests or the Wilcoxon signed-rank test were used to compare the pre-ADC, post-ADC, ΔADC, and percentage of ADC increase for *b* = 800 and *b* = 1000 acquisitions. The diagnostic performance of these potential predictors of response to N-CRT was assessed by receiver operating characteristic curve (ROC). The area under the ROC curve (AUC) was also obtained. Cutoff values, sensitivity, specificity, positive predictive value (PPV), and negative predictive value (NPV) were determined. All statistical analyses were performed using the SPSS statistical package (version 21.0; SPSS Inc., Chicago, IL, USA). *P* < 0.05 was considered to indicate a statistically significant difference.

## 3. Results

**Figure 1:**
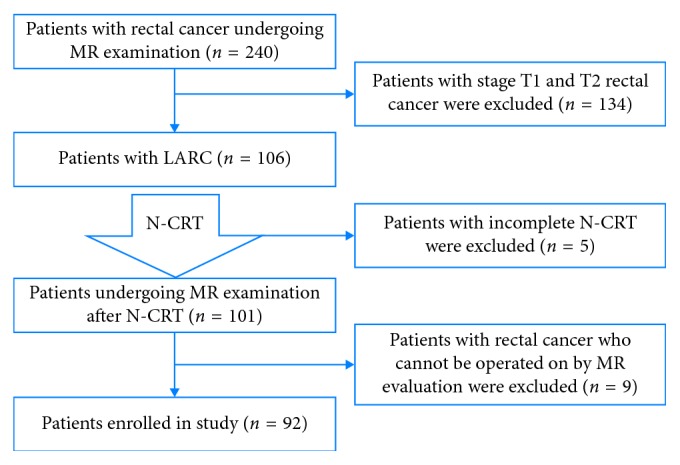
Flowchart of patient enrollment.

**Figure 2:**
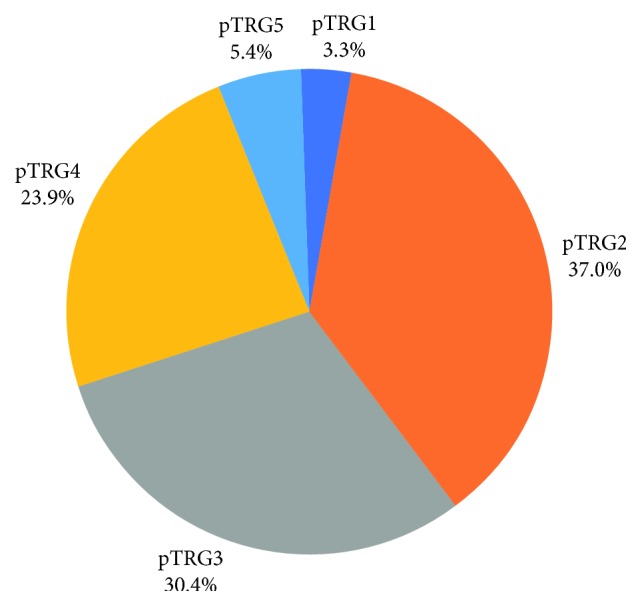
pTRG grading of the enrolled patients.

**Figure 3:**
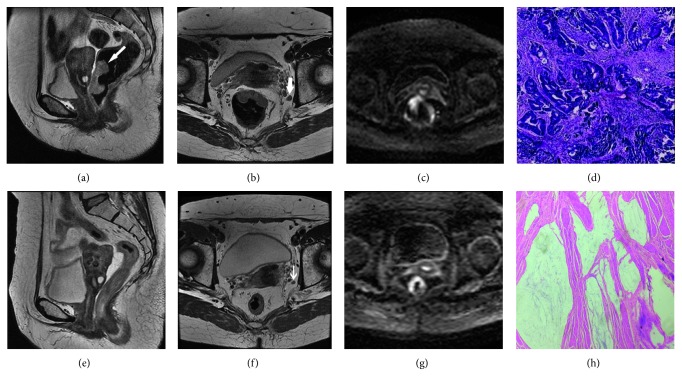
A 61-year-old woman with hematochezia in her stool for six months. (a–c) Baseline MR images before neoadjuvant therapy. (f–h) MR images after neoadjuvant therapy. (a) T2-weighted sagittal MR image shows irregular masses in the anterior rectal wall, mainly growing into the lumen. (b) Transverse-axis T2-weighted image shows irregular masses in the anterior rectal wall and enlarged lymph nodes (white arrows) outside the left mesorectal fascia. (c) Transverse and axial DWI images show the anterior rectal wall mass to be hyperintense. (e–g) The anterior rectal wall mass was significantly smaller than the anterior rectal wall mass on sagittal and axial images, corresponding to a slight thickening of the rectal wall. (d) Pathological images of the neoadjuvant anterior rectal biopsy show moderately differentiated adenocarcinoma with some mucoid changes (200x, hematoxylin and eosin (H&E) staining). (h) Postoperative pathological image of rectal cancer shows no tumor cells in the submucosa under the microscope and the presence of many mucus lakes in the submucosa and muscular layer. There were no epithelial elements in the mucus lakes, interstitial fibrosis, or chronic inflammatory cell infiltrations. A pTRG grade of 1 was given, which was in accordance with the change after radiotherapy and chemotherapy (200×, H&E staining).

A total of 92 patients with LARC were enrolled in the study (the patient enrollment process is illustrated in Figure 1). The clinical characteristics of the 92 patients and their pathological responses are summarized in Table 1.  Figure 2 reveals the postoperative pTRG results. According to histological diagnosis of the surgical specimens, 3 (3.3%) of 92 tumors were pTRG1 (Figure 3), and pTRG scores of 2, 3, 4, and 5 were recorded in 34, 28, 22, and 5 patients, respectively (Figure 3). The good responder group (pTRG1-2) contained 37/92 (40.2%) patients and the poor responder group (pTRG3–5) contained 55/92 (59.8%) patients.

**Figure 4:**
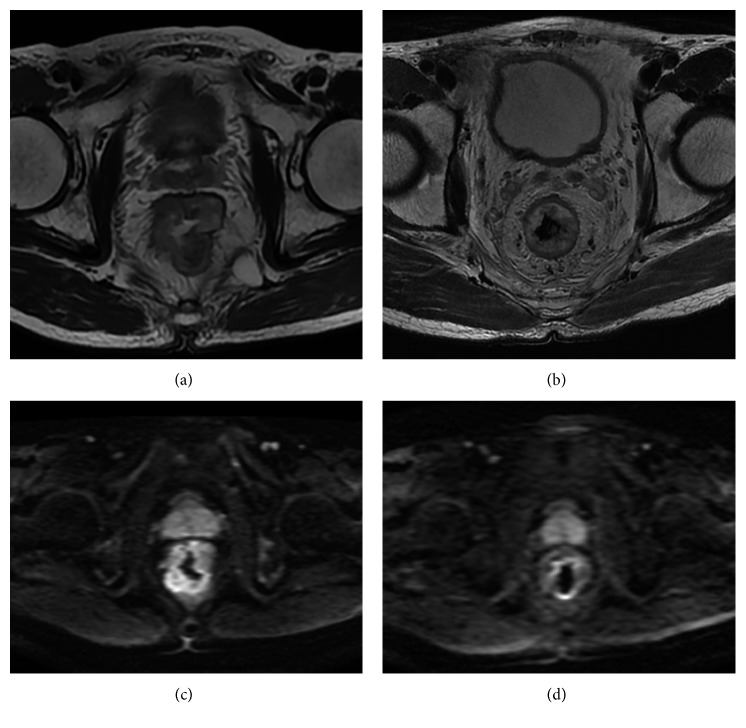
A 68-year-old man with bloody stool for more than 2 months. (a, b) Baseline conventional MRI before treatment. (c, d) Conventional MR images after treatment. (a) HR-T2WI transverse axial image shows marked uneven thickening of the rectal wall. (b) Axial DWI image shows increased DWI signal of the thickened intestinal wall, with an ADC value of 0.82 × 10^−3^ mm^2^/s. (c) HR-T2WI transverse axial image shows edema of the rectal wall and increased signal intensity. (d) Axial DWI image shows that the DWI signal of the intestinal wall is significantly lower than before, with an ADC value of 1.11 × 10^−3^ mm^2^/s.

Both before and after N-CRT, the mean ADC value for *b* = 1000 was higher than that for *b* = 800 (Figure 4(a) and 4(b)). The mean ADC values for *b* = 800 after N-CRT was significantly higher than those before N-CRT (0.992 ± 0.250×10^−3^ mm^2^/s vs. 1.270 ± 0.344×10^−3^ mm^2^/s, *P* < 0.001); the mean ADC values for *b* = 1000 after N-CRT were significantly higher than those before N-CRT (1.196 ± 0.173×10^−3^ mm^2^/s vs. 1.601 ± 0.278 ×10^−3^ mm^2^/s, *P* < 0.001).

Pre-ADC, post-ADC, ΔADC, and percentage of ADC increase for *b* (0, 800) and *b* (0, 1000) are summarized in Table 2. In the two groups, percentage of ADC increase for both *b* (0, 800) and *b* (0, 1000) showed no significant difference.

**Figure 5:**
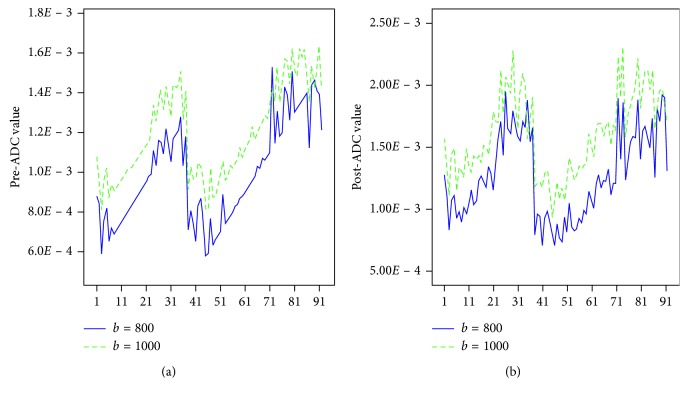
(a) Pre-ADC and (b) post-ADC values of the enrolled patients.

**Figure 6:**
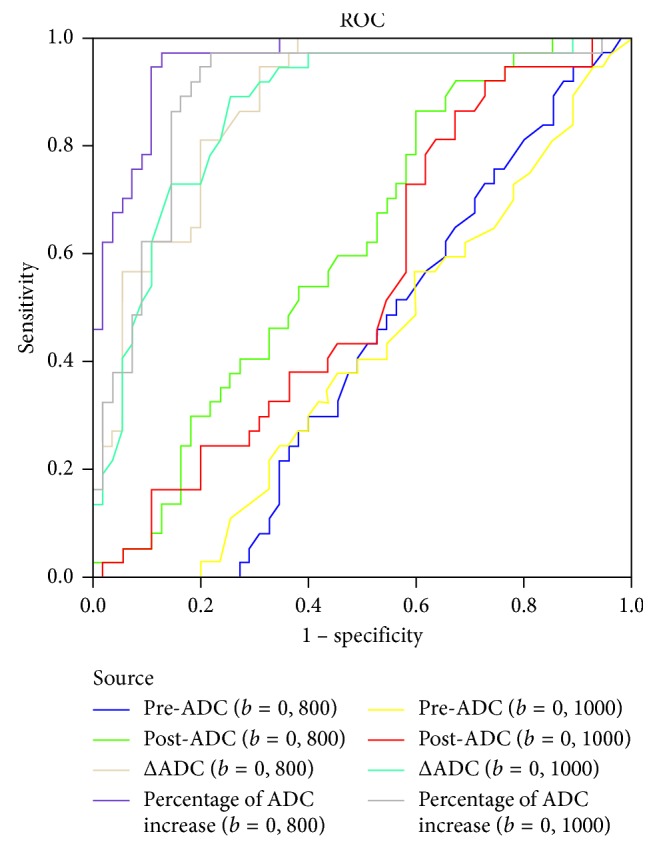
ROC curves.

To determine cutoff values and subsequently specificity, positive predictive value (PPV), and negative predictive value (NPV), Mandard's tumor regression grading protocol was considered as the reference standard for differentiating good responders from poor responders. The receiver operating characteristic (ROC) curves are shown in Figure 5, and the diagnostic performances of the ADC-related parameters are presented in Table 3. The area under the curve (AUC) for the percentage of ADC increase at *b* = 800 was 0.957, and with a cutoff value of 29.10% to differentiate the two groups of patients, the following diagnostic predictive values were observed: sensitivity, 87%; specificity, 91%; PPV, 69%; and NPV, 76%. The AUC for the percentage of ADC increase at *b* = 1000 was 0.893, and with a cutoff value of 28.67% to differentiate the two groups of patients, the sensitivity was 83%, specificity 86%, PPV 51%, and NPV 67% (Figure 6).

## 4. Discussion

For patients with LARC, N-CRT followed by surgery is a crucial standard of care with documented benefits such as local tumor control [20]. Developments in N-CRT have increased the rate of pCR, which varies between 3% and 30% in published articles [21]. It is important to determine whether patients respond well to N-CRT. For patients with a good response to N-CRT, the course of N-CRT should be prolonged and aimed at increasing the rate of pCR. A pCR suggests the possibility of less invasive treatment regimens [22]. However, N-CRT strategies may show insufficiency in certain patients with LARC, and accurate prediction of the response to N-CRT is essential to correctly perform a patient-tailored treatment strategy.

Tumor size, including tumor diameter and tumor volume, was previously used to predict the response of LARC to N-CRT. De Felice et al. reported that patients with a tumor diameter ≤5 cm were more likely to achieve pCR after N-CRT (OR 0.25; *P* value 0.035) [23], and a study conducted by Liu et al. revealed that primary tumor volume can be considered as an independent predictor of pCR (*P* value 0.036) [24]. However, in a study performed by Birlik et al. [19] that measured the tumor volumes of 41 patients with locally advanced rectal cancer before and after treatment, there was no difference in pre-N-CRT tumor volume between the good responder and poor responder groups. These conflicting results reveal that primary tumor size may be unreliable in predicting the response of LARC to N-CRT. Furthermore, the anatomical location of a tumor within the rectum is not useful for predicting pCR after neoadjuvant therapy [25].

Although several articles reported that the percentage of tumor volume regression was useful for assessing the response of rectal cancer to N-CRT [26–28], this raises some questions. First, the calculation of percentage of tumor volume regression is time dependent, and the two tumor volume measurements need plenty of time. Second, the tumor volume after N-CRT is difficult to accurately measure owing to the edema and fibrosis of the tumor that accompany N-CRT.

The response to N-CRT given by the pTRG classification is an important prognostic factor, but it is only obtained after surgical resection. With the increasing use of N-CRT for LARC, a simpler way to predict the response of LARC to N-CRT is urgently needed, and several MRI-based studies have tried to preoperatively predict the response to N-CRT [14, 29].

DWI, as a noninvasive imaging technique based on the Brownian movement of water molecules, has been used in the diagnosis of early rectal cancer [18]. The DWI-derived ADC is a quantitative parameter that can reflect histological changes in intratumoral characteristics after N-CRT [30]. ADC values are mainly negatively related to cell density and positively related to extracellular space. Several articles have suggested that quantitative analysis of ADC can serve as a biomarker for evaluating the efficacy of N-CRT for LARC [31–33]. Against this background, we aimed to identify the best DWI predictors of a good response to N-CRT in patients with LARC.

The value of pre-ADC for evaluating the response to N-CRT is highly uncertain. Kim et al. measured the ADC tumor values of 34 patients with LARC before treatment but did not find a statistically significant difference between the good responders and poor responders [34]. However, several published articles have shown conflicting results regarding the contribution of pre-ADC to the prediction of response to N-CRT [13, 15]. In those studies, pre-ADC showed a significant difference between the good responder and poor responder group. In our study, the AUC values were 0.419 for pre-ADC (*b* = 800) and 0.411 for pre-ADC (*b* = 1000). These values reveal that pre-ADC showed insufficient diagnostic performance for discriminating between good and poor responders after treatment. We speculate that individual differences in the response to N-CRT may be responsible for these conflicting results.

In previous studies, the ADC value after N-CRT was considered a more reliable predictor of tumor response than that before N-CRT [17, 35]. Our data are in agreement with these previous findings. In our study, the post-ADC AUCs were 0.692 for *b* = 800 and 0.737 for *b* = 1000, showing good diagnostic performance in assessment of the response to N-CRT. Intratumoral histological changes after N-CRT, such as reduced tumor cellularity and larger extracellular spaces, can be directly revealed by post-ADC values.

Cutoff values for post-ADC (ranging from 0.98 to 1.42 × 10^−3^ s/mm^2^) were reported to be useful for distinguishing between good responders and poor responders based on the ROC curve analysis [13]. Our data showed that a post-ADC value of 1.30 × 10^−3^ s/mm^2^ for *b* = 1000 was useful for discriminating between the good and poor responder groups, and this result is consistent with previous studies. However, in the same patient group with the same pTRG criteria, a post-ADC cutoff of 1.010 × 10^−3^ s/mm^2^ was determined for *b* = 800. Birlik at al. reported a similar result [19], with post-ADC cutoff values for discriminating between good and poor responders varying according to *b*-value within the same patient group, and the post-ADC cutoff value for *b* = 1000 was higher than that for *b* = 600. Differences in cutoff values for discriminating between good and poor responders are probably related to the different *b*-value combinations used in previous studies.

The study of Birlik et al. did not analyze whether the percentage of ADC increase showed differences between *b* = 600 and *b* = 1000 in the two groups. We compared the percentages of ADC increase between *b* = 800 and *b* = 1000 and found that the percentage of ADC increase showed no significant difference between *b* = 800 and *b* = 1000 in either the good or poor responder group. The percentage of ADC increase cutoff values for discriminating between good and poor responders was determined as 28.67% for *b* = 1000, which had a sensitivity of 88.4% and specificity of 67.1%, and 29.1% for *b* = 800, with a sensitivity of 89.1% and specificity of 73.4%. Therefore, the percentage of ADC increase may be a useful tool for discriminating between good and poor responders, while avoiding the impact of different *b*-value combinations on the prediction of response to N-CRT.

Furthermore, using ROC analysis, the AUC value for the percentage of ADC increase was 0.957 for *b* = 800 and 0.893 for *b* = 1000. Compared with the percentage of ADC increase, the AUC values for post-ADC and ΔADC were lower.

Pathological biopsy by colonoscopy showed limited value in the evaluation of response to N-CRT. It was reported that the accuracy of pathological biopsy by colonoscopy for assessment of the response to N-CRT varied between 20% and 30% [36]. Biopsy forceps used in colonoscopy are soft, and tissue specimens can only be obtained on the surface of the tumor because of the risk of several complications, such as enterobrosis and hemorrhage, especially after N-CRT. Therefore, the accuracy of pathological biopsy by colonoscopy is low.

Our study is subject to a number of limitations. First, in cases with pCR or near pCR, it was difficult to draw the ROI for ADC measurements. Second, single-slice ROIs were used for the ADC measurements rather than whole-volume analysis. However, for patients with small-size residual tumors after N-CRT, it is hard to perform whole-volume analysis on low spatial resolution ADC maps. Third, our study involved a small sample, and a multicentric study may be needed.

In conclusion, our data suggest that percentage of ADC increase, as an optimized predictor unaffected by different *b*-values, may have a significant role in predicting the response to neoadjuvant chemoradiotherapy in locally advanced rectal cancer. Additionally, post-ADC values for patients with LARC could be considered as a suitable indicator for the prediction of response to N-CRT. However, the influence of different *b*-value combinations on ADC measurements should be considered when evaluating the response to N-CRT. Accurate preoperative prediction of the response of LARC to N-CRT is necessary for further treatment strategies. Our data show that DWI with ADC may potentially help surgeons screen for patients with LARC who respond well to N-CRT and help in establishing precise patient-tailored treatments.

## Figures and Tables

**Table 1:** Clinical characteristics of the 92 patients and associations with pathological response group.

	Total (*n* = 92)	Good responder group (*n* = 37)	Poor responder group (*n* = 55)	*P* value
Age (years)	63.78 ± 11.34	65.92 ± 7.94	62.72 ± 7.89	0.061
Gender, *n* (%)				
Men	61 (66.3)	26 (70.3)	39 (70.9)	0.947
Women	31 (33.7)	11 (29.7)	16 (29.1)	
Type of surgery, *n* (%)				0.014
LAR	9 (9.8)	7 (18.9)	2 (3.6)	
ISR	7 (7.6)	2 (5.4)	5 (9.1)	
Local resection	2 (2.2)	2 (5.4)	0 (0)	
Dixon	52 (56.5)	22 (59.5)	30 (54.5)	
Hartmann	4 (4.3)	0 (0)	4 (7.3)	
APR	18 (19.6)	4 (10.8)	14 (25.5)	
Pathological T stage, *n* (%)				<0.001
T0	3 (3.3)	3 (8.1)	0 (0)	
T1-2	44 (47.8)	26 (70.3)	17 (30.9)	
T3	41 (44.6)	7 (18.9)	35 (63.6)	
T4	4 (4.3)	1 (2.7)	3 (5.5)	

**Table 2:** Comparison of ADC values between *b* (0, 800) and *b* (0, 1000).

	*b* (0, 800)	*b* (0, 1000)	*P* value
Good responder group			
Pre-ADC value (×10^−3^ mm^2^/s)	0.91 (0.78–1.12)	1.10 (0.98–1.35)	0.012
Post-ADC value (×10^−3^ mm^2^/s)	1.28 (1.07–1.62)	1.57 (1.39–1.85)	0.031
ΔADC (×10^−3^ mm^2^/s)	0.40 (0.28–0.52)	0.46 (0.38–0.52)	0.003
Percentage of ADC increase	0.422 ± 0.090	0.408 ± 0.101	0.569
Poor responder group			
Pre-ADC value (×10^−3^ mm^2^/s)	0.98 (0.80–1.31)	1.20 (1.02–1.44)	0.025
Post-ADC value (×10^−3^ mm^2^/s)	1.20 (0.93–1.54)	1.61 (1.27–1.86)	0.041
ΔADC (×10^−3^ mm^2^/s)	0.16 (0.11–0.26)	0.21 (0.11–0.34)	0.011
Percentage of ADC increase	0.188 ± 0.0930	0.194 ± 0.126	0.741

**Table 3:** Diagnostic performance of ADC-related parameters.

	AUC (95% CI)	Cutoff value	Sensitivity (%)	Specificity (%)	PPV (%)	NPV (%)
Pre-ADC (*b* = 800)	0.419 (0.304–0.534)	0.87 × 10^−3^ mm^2^/s	33	41	31	43
Post-ADC (*b* = 800)	0.692 (0.496–0.724)	1.01 × 10^−3^ mm^2^/s	41	65	51	64
ΔADC (*b* = 800)	0.879 (0.811–0.946)	0.232 × 10^−3^ mm^2^/s	79	72	66	53
Percentage of ADC increase (*b* = 800)	0.957 (0.92–0.993)	29.10%	87	91	69	76
Pre-ADC (*b* = 1000)	0.411 (0.295–0.527)	1.03 × 10^−3^ mm^2^/s	41	37	41	45
Post-ADC (*b* = 1000)	0.537 (0.418–0.655)	1.30 × 10^−3^ mm^2^/s	43	64	74	47
ΔADC (*b* = 1000)	0.866 (0.789–0.942)	0.335 × 10^−3^ mm^2^/s	74	56	47	69
Percentage of ADC increase (*b* = 1000)	0.893 (0.822–0.965)	28.67%	83	86	51	67

CI: confidence interval; PPV: positive predictive value; NPV: negative predictive value.

## Data Availability

The data used to support the findings of this study are available from the corresponding author upon request.
